# Cell Free DNA of Tumor Origin Induces a ‘Metastatic’ Expression Profile in HT-29 Cancer Cell Line

**DOI:** 10.1371/journal.pone.0131699

**Published:** 2015-07-02

**Authors:** István Fűri, Alexandra Kalmár, Barnabás Wichmann, Sándor Spisák, Andrea Schöller, Barbara Barták, Zsolt Tulassay, Béla Molnár

**Affiliations:** 1 2^nd^ Dept. of Internal Medicine, Semmelweis University, Budapest, Hungary; 2 Hungarian Academy of Sciences, Molecular Medicine Research Unit, Budapest, Hungary; University of Navarra, SPAIN

## Abstract

**Background:**

Epithelial cells in malignant conditions release DNA into the extracellular compartment. Cell free DNA of tumor origin may act as a ligand of DNA sensing mechanisms and mediate changes in epithelial-stromal interactions.

**Aims:**

To evaluate and compare the potential autocrine and paracrine regulatory effect of normal and malignant epithelial cell-related DNA on TLR9 and STING mediated pathways in HT-29 human colorectal adenocarcinoma cells and normal fibroblasts.

**Materials and Methods:**

DNA isolated from normal and tumorous colonic epithelia of fresh frozen surgically removed tissue samples was used for 24 and 6 hour treatment of HT-29 colon carcinoma and HDF-α fibroblast cells. Whole genome mRNA expression analysis and qRT-PCR was performed for the elements/members of TLR9 signaling pathway. Immunocytochemistry was performed for epithelial markers (i.e. CK20 and E-cadherin), DNA methyltransferase 3a (DNMT3a) and NFκB (for treated HDFα cells).

**Results:**

Administration of tumor derived DNA on HT29 cells resulted in significant (p<0.05) mRNA level alteration in 118 genes (logFc≥1, p≤0.05), including overexpression of metallothionein genes (i.e. *MT1H*, *MT1X*, *MT1P2*, *MT2A*), metastasis-associated genes (i.e. *TACSTD2*, *MACC1*, *MALAT1*), tumor biomarker (CEACAM5), metabolic genes (i.e. *INSIG1*, *LIPG*), messenger molecule genes (i.e. *DAPP*, *CREB3L2*). *Increased* protein levels of CK20, E-cadherin, and DNMT3a was observed after tumor DNA treatment in HT-29 cells. Healthy DNA treatment affected mRNA expression of 613 genes (logFc≥1, p≤0.05), including increased expression of key adaptor molecules of TLR9 pathway (e.g. *MYD88*, *IRAK2*, *NFκB*, *IL8*, *IL-1β*), STING pathway (ADAR, IRF7, CXCL10, CASP1) and the *FGF2* gene.

**Conclusions:**

DNA from tumorous colon epithelium, but not from the normal epithelial cells acts as a pro-metastatic factor to HT-29 cells through the overexpression of pro-metastatic genes through TLR9/MYD88 independent pathway. In contrast, DNA derived from healthy colonic epithelium induced TLR9 and STING signaling pathway in normal fibroblasts.

## Introduction

Altered epithelial-stromal interactions are fundamental in cancer formation. Among the well-known regulatory ligands (e.g. growth factors, cytokines, chemokines, sex hormones) tumor tissue-derived DNA is also involved in this communication via cellular receptors sensing DNA [1] According to several studies [2–4] the DNA fragments of tumor origin (i.e. 21 to 500 bases short sequences of human origin) play a role in the formation of a tumor supportive microenvironment (i.e. promote tumor invasion and evasion of immune surveillance) [5–8] The cell free DNA originates from necrotic/apoptotic tumor cells, and can be actively released by living cells to the intercellular compartment [9–12]. This tumor tissue originated DNA is detectable in the plasma and serum and could serve a useful biomarker for cancer detection [11]. It contains a number of cancer specific entities, including oncogenes, tumor suppressor genes, aberrant microsatellites, aberrant DNA methylation genes, and rearranged chromosomal DNA [12]. Recent studies confirmed the uptake and the retention of oncogenes stimulating cell proliferation in non-malignant cells after integration (oncometastasis) into the recipients cell´s genome [13].

To the extent they are understood, the DNA sensing mechanisms in the target cells comprise two primary adaptor pathways, i.e. toll-like receptor (TLR9) and the stimulator of interferon genes (STING) pathways.[14] Cytoplasmic TLR9 recognizes endogenous ligands, such as danger-associated molecular patterns (DAMPs) like unmethylated DNA sequences [7, 8, 15] The total amount of unmethylated DNA increases in parallel with global DNA hypomethylation in tumor tissue compared to the normal tissue [16]. The increased expression of TLR9 was detected in several tumor types.[1, 17–20] Increased TLR9 expression in carcinoma cells was associated with higher metastatic potential, while higher TLR9 expression by fibroblast-like cells was associated with a low probability of metastasis [21]

The STING signaling pathway is an adaptor for DNA via binding of cyclic dinucleotides generated by the enzyme cyclic GMP-AMP (cGAMP) synthase (cGAS).[22–24]

Strong synergism has been observed among cooperating STING and TLR9 signaling. These two signaling pathways are differentially regulated by crucial adaptor molecules (IRF3/7, STING, and MyD88) [25]. Furthermore Deng et al. (2014) and Woo et al. (2014) provided evidence suggesting dendritic cells detect DNA from tumor cells via the STING-mediated, cytosolic DNA sensing pathway. [22, 26, 27]

Based on our previous results, HT-29 human colon adenocarcinoma cells reflected altered DNA methylation level (via elevated DNMT3a methyltranferase activity) and CK20 epithelial marker expression after re—administration of self DNA.[28] In the present study we analyzed the autocrine and paracrine effects of DNA from tumor and healthy tissue on HT-29 cancer cells and fibroblasts by whole genomic mRNA expression analysis, and qRT PCR for validation of genes from TLR9 pathway. Furthermore immunocytochemical analysis was performed for selected differentiation markers, cell- adhesion molecules, and methyltransferases, for verification at the protein level following treatment with DNA from healthy and tumor tissues.

## Materials and Methods

### HT-29 cell culture

HT-29 human colon adenocarcinoma cells were purchased from LGC STANDARDS (cat. No. ATCC HTB-38) and cultured in a specific pathogen-free cell culture laboratory at 37°C in 5% CO_2_. HT-29 cells were maintained in McCoy's 5a Medium Modified (Cat No. M8403-500 mL Sigma-Aldrich, St Louis, USA) supplemented with 10% (vol/vol) fetal bovine serum (FBS; Standard Quality; PAA Laboratories GmbH, Pasching, Austria), 160 μg/ml gentamycin (Sandoz, Sandoz GmbH, Austria), and 125 μg/ml amphotericin B (Sigma-Aldrich, St Louis, USA).

### HDFα cell culture

HDFα cells were purchased from Life technologies (cat. No. C0135C) and cultured in a specific pathogen-free cell culture laboratory at 37°C in 5% CO2. HDFα cells were maintained in Medium 106 (Life Technologies Corporation, Carlsbad, USA) supplemented with LSGS (Life Technologies Corporation, Carlsbad, USA) and amphotericin B (Sigma). 160 μg/ml gentamycin (Sandoz, Sandoz GmbH, Austria).

### DNA isolation

Genomic DNA was isolated from macroscopically normal areas near colorectal cancers (CRC) and from the tumorous tissue areas of surgically removed macrodissected fresh frozen CRC samples (stage II, moderately differentiated tumors from sigmoid colon and rectum (25–50 mg tissue) by High Pure PCR template preparation kit (Roche GmbH, Germany). These CRC patients were all confirmed by definitive histological diagnosis and received no preoperative chemotherapy or radiotherapy. The isolated, protein-free DNA was treated with 20 μl RNase A/T1 Mix at 37°C for 1 hour (Thermo Scientific, Germany). The concentration of isolated and purified DNA was determined by Nanodrop-1000 spectrophotometer (Thermo Scientific, Germany).

### Ethics statement

The study was conducted according to the declaration of Helsinki and approved by Semmelweis University Ethics Committee and the governmental Regional and Institutional Committee of Science and Research Ethics (TUKEB), Nr: 23970-2/2011). Written informed consent was obtained from all patients included in the study.

### DNA treatment of HT-29 and HDFα cells

HT-29 and HDFα cells were seeded (at a density of 0.5x10^6^ and 0.1x10^6)^ into 6 well Corning CellBIND cell culture multiwell plates in RPMI 1640 supplemented with gentamycin, amphotericin B and FBS. When monolayers reached 80–90% of confluency, 15 μg of normal or tumor DNA (dissolved in 200 μl sterile phosphate buffered saline (PBS) were added to the wells. The negative control comprised appropriate volumes of PBS. Cells were incubated at 37°C in a 5% CO2 atmosphere and 95% humidity.

### Isolation of total RNA for Affymetrix HGU133 Plus 2.0 microarray and qRT-PCR gene expression analyses

After 24 hours, cells were washed two times in 5 ml sterile PBS. After the second wash, cells were resuspended in 5 ml PBS. 2.5 ml of cell suspension was used for total RNA isolation. Total RNA from the isolated HT-29 cells was extracted with the RNeasy Mini kit (Qiagen, USA) according to the instructions of the manufacturer. The isolated RNA was stored at -80°C.

### mRNA expression microarray analysis

The quantity and quality of the isolated RNA were tested by measuring absorbance and by capillary gelelectrophoresis using the 2100 Bioanalyzer and RNA 6000 Pico Kit (Agilent Inc, Santa Clara, US). Biotinylated cRNA probes were synthesized from 4.82±0.60 μg total RNA and fragmented using the One-Cycle Target Labeling and Control Kit according to the Affymetrix description. Ten μg of each fragmented cRNA sample were hybridized into HGU133 Plus2.0 array (Affymetrix) at 45°C for 16 hours. Microarrays were washed and stained using Fluidics Station 450 (Affymetrix) and an antibody amplification staining method according to the manufacturer's instructions. The fluorescent signals were detected by a GeneChip Scanner 3000 (Affymetrix).

### Statistical evaluation of mRNA expression profiles

Pre-processing and quality control analyses were performed according to the suggestions of The Tumour Analysis Best Practices Working Group (Tumor Analysis Best Practices Working Group, 2004). Scanned images were inspected for artifacts; the percentage of present calls (>25%) and degree of RNA degradation were evaluated. On the basis of evaluation criteria, all measurements fulfilled the minimal quality requirements. In the case of HT29 and HDFα cell experiments, the similarity of the 2–2 biological replicates was stated using the Euclidean distance method. Affymetrix expression arrays were pre-processed by gcRMA with quantile normalization and median polish summarization.

Further analyses to identify differentially expressed features was performed with significance analysis of microarrays (SAM). The nearest shrunken centroid method (prediction analysis of microarrays = PAM) was applied for sample classification from gene expression data. Prediction analysis of microarrays uses soft thresholding to produce a shrunken centroid, which allows the selection of characteristic genes with high predictive potential (Tibshirani et al, 2002). Pre-processing, data mining and statistical steps were performed using Bioconductor libraries in the R-environment. Annotation and functional classification of discriminatory genes were performed using the Affymetrix NetAffx system.

### Reverse transcription and quantitative real time polymerase chain reaction

After quantitative (Nanodrop) and qualitative analysis (BioAnalyzer RNA 6000 Pico Kit chip kit RNA program; RIN>8 in all cases), reverse transcription was performed by using 1 μg of total RNA (High Capacity cDNA Reverse Transcription Kit, Applied Biosystems, USA).

Quantitative real-time (qRT) PCR was performed using Probes Master and SYBR green (Roche GmbH, Germany). Gene expression levels for each individual sample were normalized to GAPDH expression. Mean relative gene expression was determined and differences were calculated using the 2-ΔC(t) method. Oligonucleotide primers of TLR 9 (MYD88-dependent) signaling pathway are listed in Table 1.

**Table 1:** List of the oligonucleotide primers of TLR9 (MYD88-dependent) signaling pathway.

Gene Symbol	Gene title	Sequence of selected primers
hTLR9_F	human toll-like receptor 9 (fwd)	CCTCCTGCTCAAGCTACACC
hTLR9_R	human toll-like receptor 9 (reverse)	CTTGTCCTTTTCTGCCCTTG
hMYD88_aF	human MYD88 (forward)	GAAGAAAGAGTTCCCCAGCA
hMYD88_aR	human MYD88 (reverse)	GTGCAGGGGTTGGTGTAGTC
hMYD88_bF	human MYD88 (forward)	CTCCTCCACATCCTCCCTTC
hMYD88_bR	human MYD88 (reverse)	CGCACGTTCAAGAACAGAGA
hIRAK2_F	human interleukin associated kinase (forward)	CTTGGAGTGGGCTTTCTGAG
hIRAK2_R	human interleukin associated kinase (reverse)	AGGCTGGAATTGTCAACGTC
hIRAK4_F	human interleukin associated kinase(forward)	GCTGCCTCAATGTTGGACTAA
hIRAK4_R	human interleukin associated kinase(reverse)	TTCCATCCTTCTTGAGGATCA
hTRAF6_F	human tumor necrosis factor receptor associated factor 6 (forward)	CTTTGGCAAATGTCATCTGTG
hTRAF6_R	human tumor necrosis factor receptor associated factor 6 (reverse)	CTGAATGTGCATGGAATTGG
hNFκB1_F	human nuclear factor κB (forward)	TATGTGGGACCAGCAAAGGT
hNFκB1_R	human nuclear factor κB (reverse)	GCAGATCCCATCCTCACAGT
hIL8_F	human interleukin 8 (forward)	GTGCAGTTTTGCCAAGGAGT
hIL8_R	human interleukin 8 (reverse)	AAATTTGGGGTGGAAAGGTT
hIL1B_F	human interleukin 1β (forward)	GCATCCAGCTACGAATCTCC
hIL1B_R	human interleukin 1β (reverse)	GCATCTTCCTCAGCTTGTCC
hDNMT1_F	human DNA methyltransferase 1 (forward)	AGGCAGTTCAACACCCTCAT
hDNMT1_R	human DNA methyltransferase 1 (reverse)	TGACGGTTGTGCTGAAGAAG
hTNFa_F	human tumor necrosis factor α (forward)	CCTGTGAGGAGGACGAACAT
hTNFa_R	human tumor necrosis factor α (reverse)	GGTTGAGGGTGTCTGAAGGA

### Cell viability assays

0.1x10^6^ HT-29 cells were seeded at a density of 0.1x10^6^ into Corning CellBIND cell culture multiwell plates in RPMI 1640, supplemented with gentamycin and FCS. After 24 hours, the medium was changed; and, 15 μg of tumor and normal DNA [dissolved in 200 μl sterile phosphate buffered saline (PBS)] was added to the wells. The negative control is comprised of an appropriate volume of PBS. Cells were incubated at 37°C in a 5% CO2 atmosphere and 95% humidity for 72h.

After 72 hour treatment, the cells were harvested, washed twice in 0.5 ml sterile PBS, re-suspended in 1.0 ml of ice cold 70% ethanol and stored at -20°C. Measurement was performed in triplicates for each treatment group.

Samples were centrifuged for 3 min at 1300 rpm. Then, the cells were re-suspended in 300 μ1 of extraction buffer; and, 3 μl of RNAse (RNase A/T1 Mix, Thermo Fisher Scientific Baltics UAB, Vilnius, Lithuania) was added. After 15 min incubation at room temperature, 3 μl of propidium jodide (Sigma-Aldrich, St. Louis, USA; Cat. No. 81845) was added. The FACS measurement was performed on BD FACScalibur (New Jersey, USA).

Trypan blue exclusion tests were performed to assess the cell viability following exposure to tumor and normal cell free DNA. The HT-29 cell´s viability was then quantified by counting the number of living and dead cells using a hemocytometer at the time point 72h post tumor and normal DNA administration.

### Immunocytochemical analysis

From the HT-29 cell suspension 2.5 ml was used for immunocytochemical (ICC) analyses. HT-29 cells were cyto-centrifuged onto a coverslip and fixed in methanol at -20°C for 10 min, and then incubated in TBS containing 1% bovine serum albumin, 10% normal goat serum, 0.3 M glycine and 0.1% Tween 20, for 1h to permeabilize the cells and block non-specific protein-protein interactions.

2x10^5^ HDFα fibroblasts were seeded to a Lab-Tek 8-well chamber slides in 500 μl media (Thermo Scientific, Rochester, NY) and cultured until 80–90% confluence. After DNA treatment the cells were fixed in ice cold methanol at -20°C for 10 min, and then incubated in PBS containing 1% bovine serum albumin, /10% normal goat serum, /0.3 M glycine and in 0.1% Tween 20, TBS-Tween for 1h to permeabilize the cells and block non-specific protein—protein interactions.

For immunostaining, cells were treated with mouse anti-human monoclonal anti-TLR9 IgG (1:300; ab85860, Abcam, Cambridge, MA, USA), and anti-DNMT3a IgG (1:300; ab13888, Abcam, Cambridge, MA, USA) at 4°C overnight, using HeLa cells as a positive control; anti-cytokeratin 20 (1:200, clone: PCK-26, Dako, Glostrup, Denmark), E-cadherin (1:2, clone: ECH6, Histopathology Ltd, Pecs, Hungary) using human colonic mucosa as a positive control; and rabbit anti-NFκB IgG (1:100, GTX102090, Genetex, San Antonio, Texas, USA) using HepG2 cells as a positive control.

Sections were incubated for 30 min with peroxidase-labeled polymer-horseradish peroxidase (HRP) conjugated to goat anti-mouse/rabbit immunoglobulins (Envision + System-HRP-DAB; Dako, Glostrup, Denmark). Staining was completed by incubation with 3,3′diaminobenzidine chromogen solution (chromogen solution is part of Envision kit). The sections were counterstained with hematoxylin.

### ICC evaluation

Slides were digitalized using a high resolution Panoramic Scan instrument (3DHistech Ltd., Hungary) and analyzed with the Panoramic Viewer Histoquant Module software (3DHistech Ltd., Hungary). 1000 cells/slide were evaluated. In the case of E-cadherin, CK20, cytoplasmic immunoreactions were scored as negative (-), weak (+), moderate (++) and strong (+++). NFκB, DNMT3a nuclear/cytoplasmic expressions were scored as negative (-), weak (+), moderate (++) and strong (+++) immunoreactions. The density of negative (-), weak (+), moderate (++) and strong positive (+++) pixels in immunoreactive cells was evaluated.

## Results

### Effect of normal and malignant epithelial DNA to HT-29 cells

**Fig 1:**
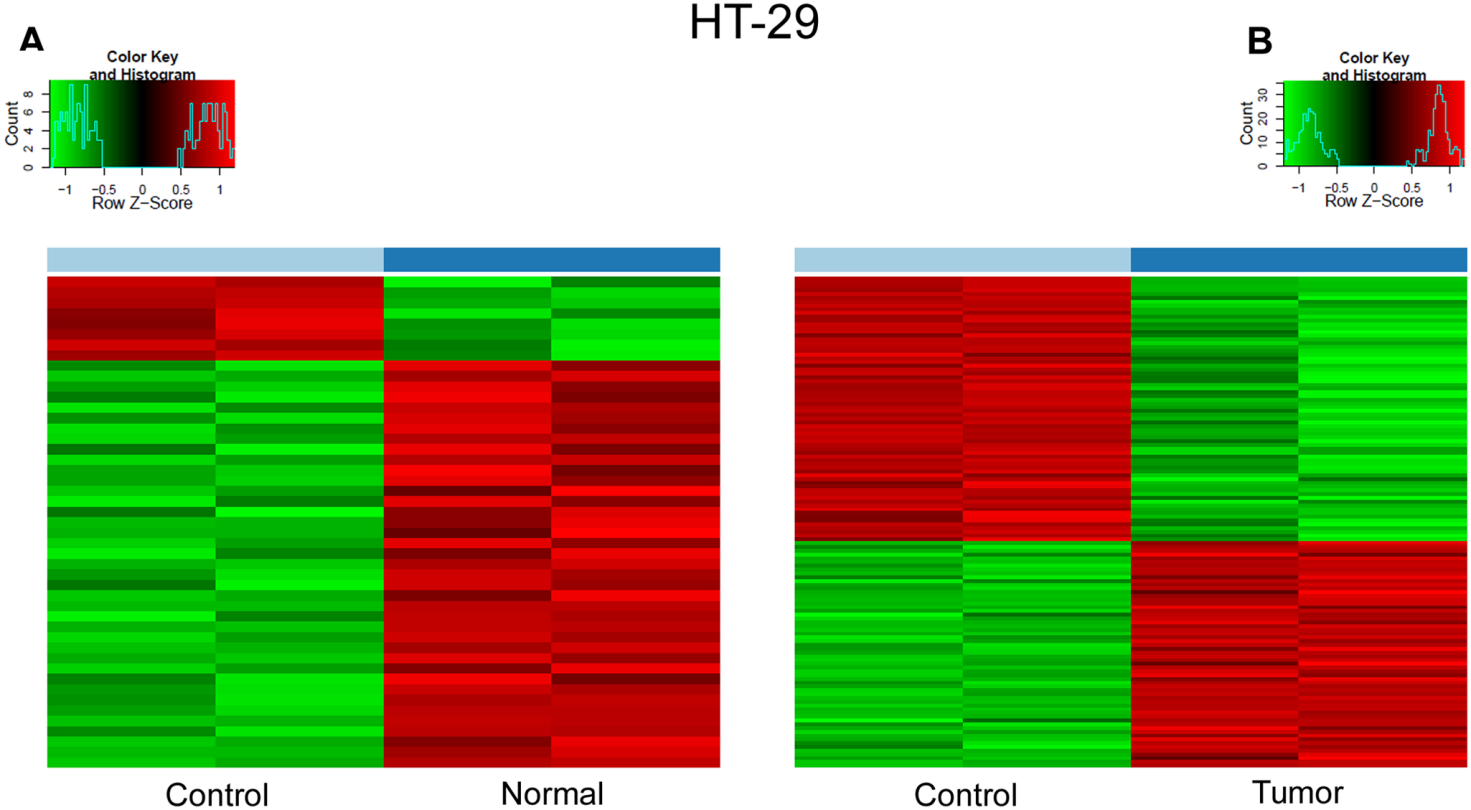
Heat map representing RNA expression changes in control HT-29 cells and HT-29 cells treated with DNA isolated from healthy(A) and tumorous(B) colonic epithelium.

First, we treated the HT-29 cells with DNA isolated from normal or tumor tissue. Both treatments induced mRNA overexpression of metallothionein genes (i.e. MT1H, MT1G, MT1X, MT1P2 and MT2A) (metallothionein 1H, -1G, -1X, -1P2, -2A) (Fig 1A and 1B). Tumor tissue derived DNA treatment resulted in significant overexpression of n = 118 genes (at the mRNA level). Among these genes, we observed metastasis associated genes e.g., tumor biomarker CEACAM5 (carcinoembryonic antigen-related cell adhesion molecule 5), metabolic genes e.g. INSIG1 (insulin induced gene 1) LIPG (endothelial lipase) and messenger molecule genes DAPP1(dual adaptor of phosphotyrosine and 3-phosphoinositides), CREB3L2 (cAMP responsive element binding protein 3-like 2) (Table 2)

**Table 2:** Significant gene expression changes after tumor tissue-originated DNA treatment in HT-29 cells.

Role in tumorigenesis	Gene symbols	References
metastasis associated genes	MACC1, MALAT1, TACSTD2	[31],[32],[29]
tumor biomarker	CEACAM5	[30]
metabolic genes	INSIG1, LIPG	[31, 32]
messenger molecule genes	DAPP, CREB3L2	[33]

A complete list of regulated genes is available at (http://www.ncbi.nlm.nih.gov/geo) with accession number GSE67557.

### Analysis of 12 elements of TLR9 pathway in HT-29 cells by qRT—PCR

**Fig 2:**
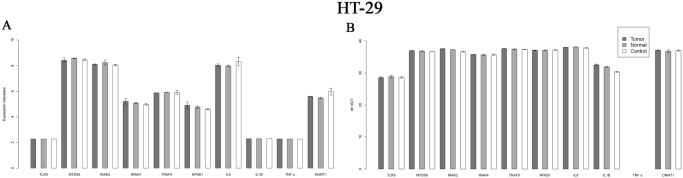
Expression changes of TLR9 MYD88 dependent pathway genes on Affymetrix U 133 2.0 microarray in control, normal and tumorous DNA treated samples(A) Figure and q RT-PCR analysis of TLR9 MYD88 dependent pathway of HT-29 cells(B).

In HT-29 cells either tumor and healthy colon tissue derived DNA significantly affected the expression of IL-1β gene related to PBS treated controls. The overexpression of this gene was higher after tumor tissue derived DNA treatment (log Fc 2.33; p≤0.05), than in samples treated with normal DNA (log Fc 1.59; p≤0.05) related to untreated controls (Fig 2A and 2B).

### Cell viability assays

**Fig 3:**
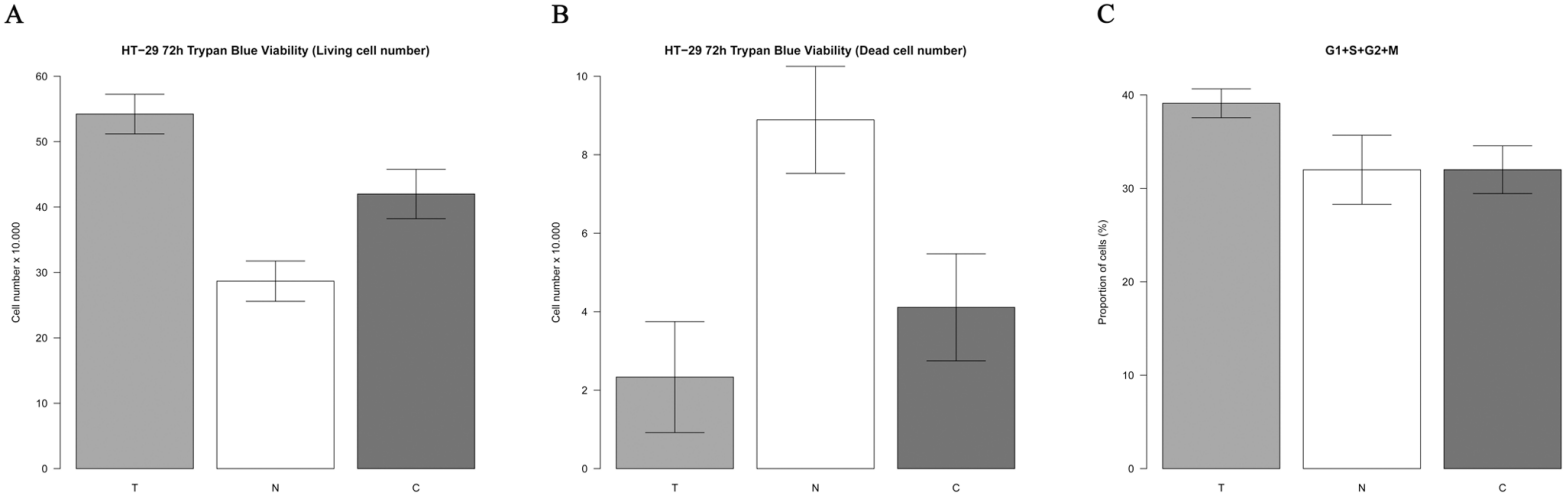
Number of living(A) and dead(B) HT-29 cells determined by Trypan blue exclusion test; PI cell-cycle analysis of HT-29 colon cancer cells(C).

The results of trypan blue exclusion test reflected that tumor cell free DNA treatment significantly increased living cell number in the relation to control group (av. 54.22±3.03 vs. 42.00±3.78; p≤0.05). We also detected significantly decreased number of living cells in normal DNA treated group related to control group (av. 28.67±3.08 vs. 42.00±3.78; p≤0.05); (Fig 3A and 3B).

The PI cell cycle analysis showed significantly higher living cell population (G1+S+G2+M phase) in tumor DNA treated group vs. control group (av. 39.11±0.57 vs. 32.00±2.50; p≤0.05). Normal DNA treatment did not affected the cell cycle of counted cells, but we observed a lower cell count in normal DNA treated samples (Fig 3C).

### Effect of normal and malignant epithelial DNA on fibroblast cells

**Fig 4:**
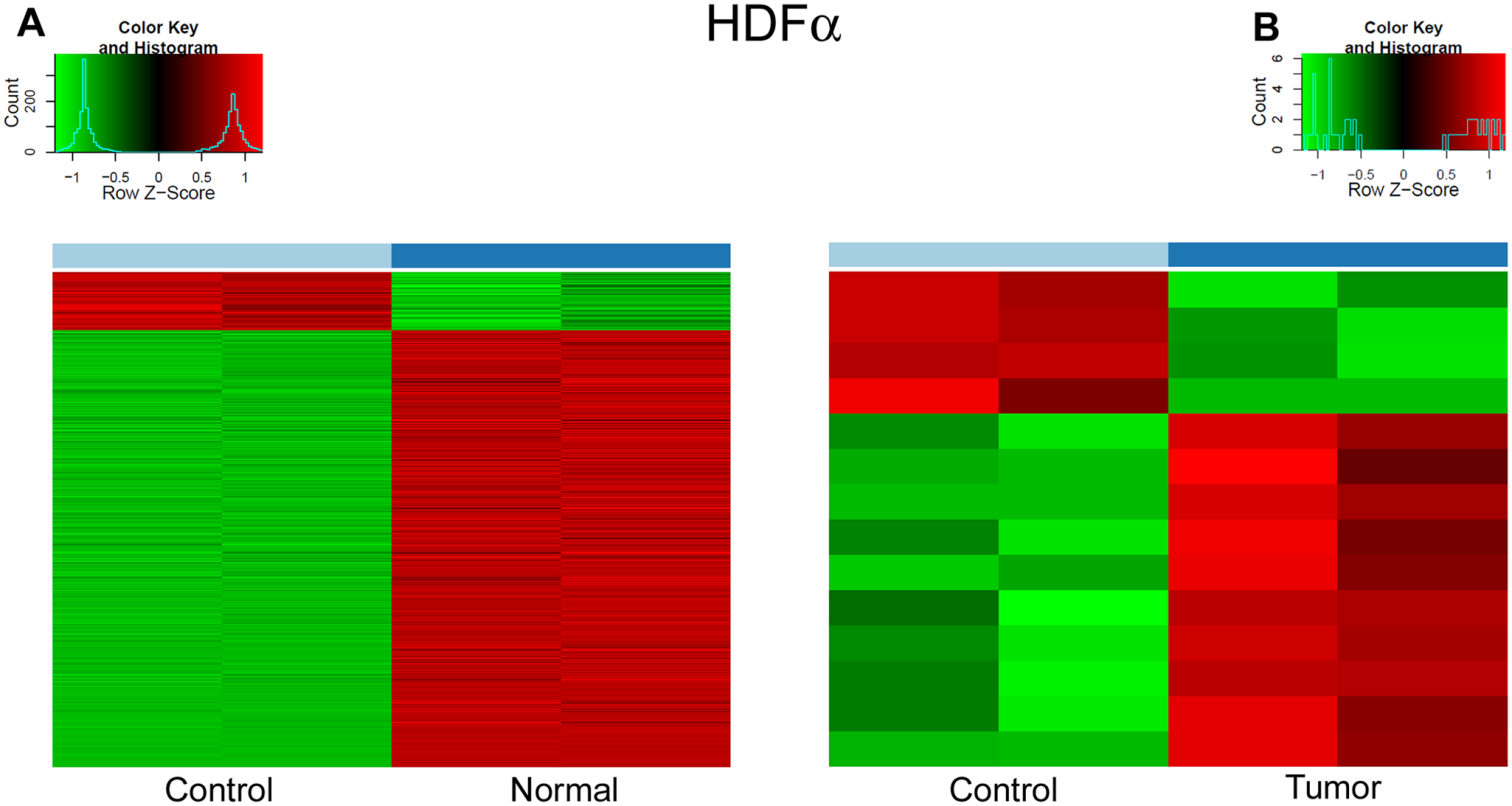
Heat map representing RNA expression changes in control HDFα cells and HDF-α cells treated with DNA isolated from healthy (A) and tumorous (B) colonic epithelium.

Treatment of HDFα fibroblasts with normal DNA resulted in overexpression of many genes in the TLR9 MYD88 pathway, cytokines, chemokines, growth factors, apoptosis related genes, and prostaglandins. In total, DNA from normal colon tissue resulted in a significant expression change for n = 613 genes (Fig 4A). In contrast, treatment with tumor tissue-derived DNA affected the expression of only n = 12 genes (Fig 4B).

Important gene expression changes induced by healthy tissue originated DNA treatment in HDFα cells are summarized in Table 3. The cell free DNA induced the upregulation of chemokine ligands as chemotactic factors, prostaglandins and receptors for prostaglandines in addition to the DNA sensing pathways.

**Table 3:** Significant gene expression changes after normal colon tissue-originated DNA treatment in HDFα fibroblasts.

Role in tumorigensis	Gene symbols	References
TLR9 MYD88 pathway	MYD88, IRAK2, TRAF6, NFκB, IL6, IL8, IRF7, IFN-β	[21]
STING/cGAS pathway	ADAR, NFκB, CASP1, CXCL10, IRF7, IFN-β, IL-6	[34]
chemokines	CCRL1, CCL8, CXCL3, CXCL5, CXCL10, CXCL11	
fibroblast growth factor	FGF2	
apoptosis related genes	CASP7,CFLAR, BCL2	
prostaglandines	PTGS2, PTGFR	

Following tumor DNA treatment of HDFα fibroblasts, we observed no overexpression of the elements of TLR9 pathway, chemokines, growth factors, apoptosis related genes, prostaglandines and receptors for prostaglandines.

A complete list of regulated genes is available at (http://www.ncbi.nlm.nih.gov/geo) with accession number GSE67557.

### Analysis of 12 elements of TLR9 pathway in fibroblasts by qRT-PCR

**Fig 5:**
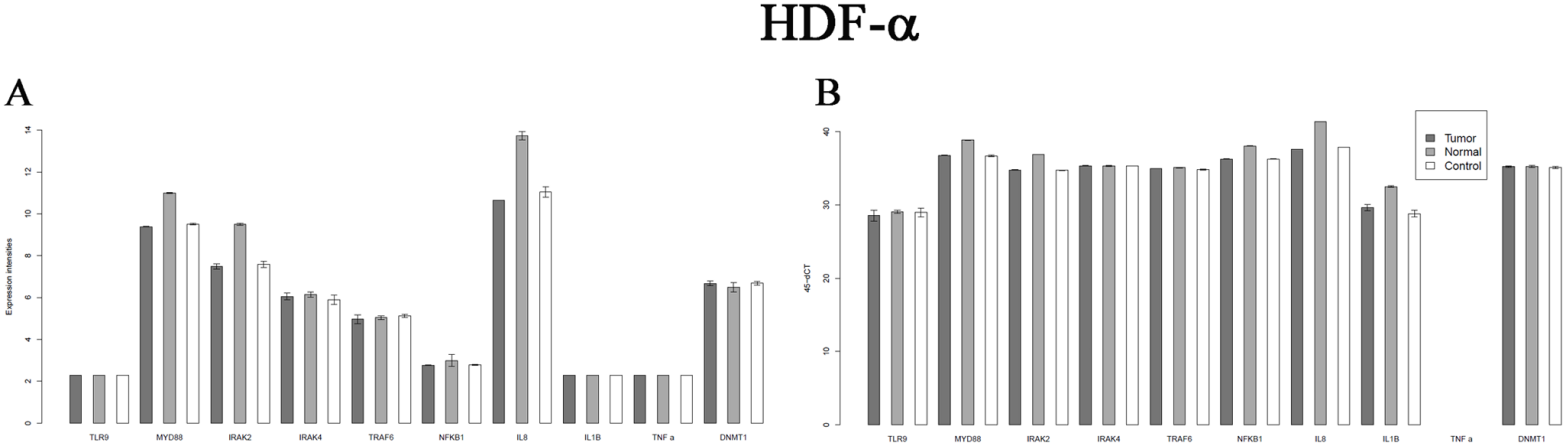
Expression changes of TLR9 MYD88 dependent pathway genes on Affymetrix U 133 2.0 microarray in control, normal and tumorous DNA treated samples(A) and q RT-PCR analysis of TLR9 MYD88 dependent pathway on HDF alpha cells(B).

Five genes of canonical TLR9 pathway (IRAK2, MYD88, NFKB, IL-8, IL-1β) showed significant overexpression after the treatment with normal tissue derived cell free DNA related to control cells. Tumor tissue derived DNA treatment did not affected the genes of TLR9 MYD88 pathway. (Log Fc ≥1; p≤0.05); (Fig 5A and 5B).

### ICC analysis of epithelial differentiation, adhesion and methylation markers

**Fig 6:**
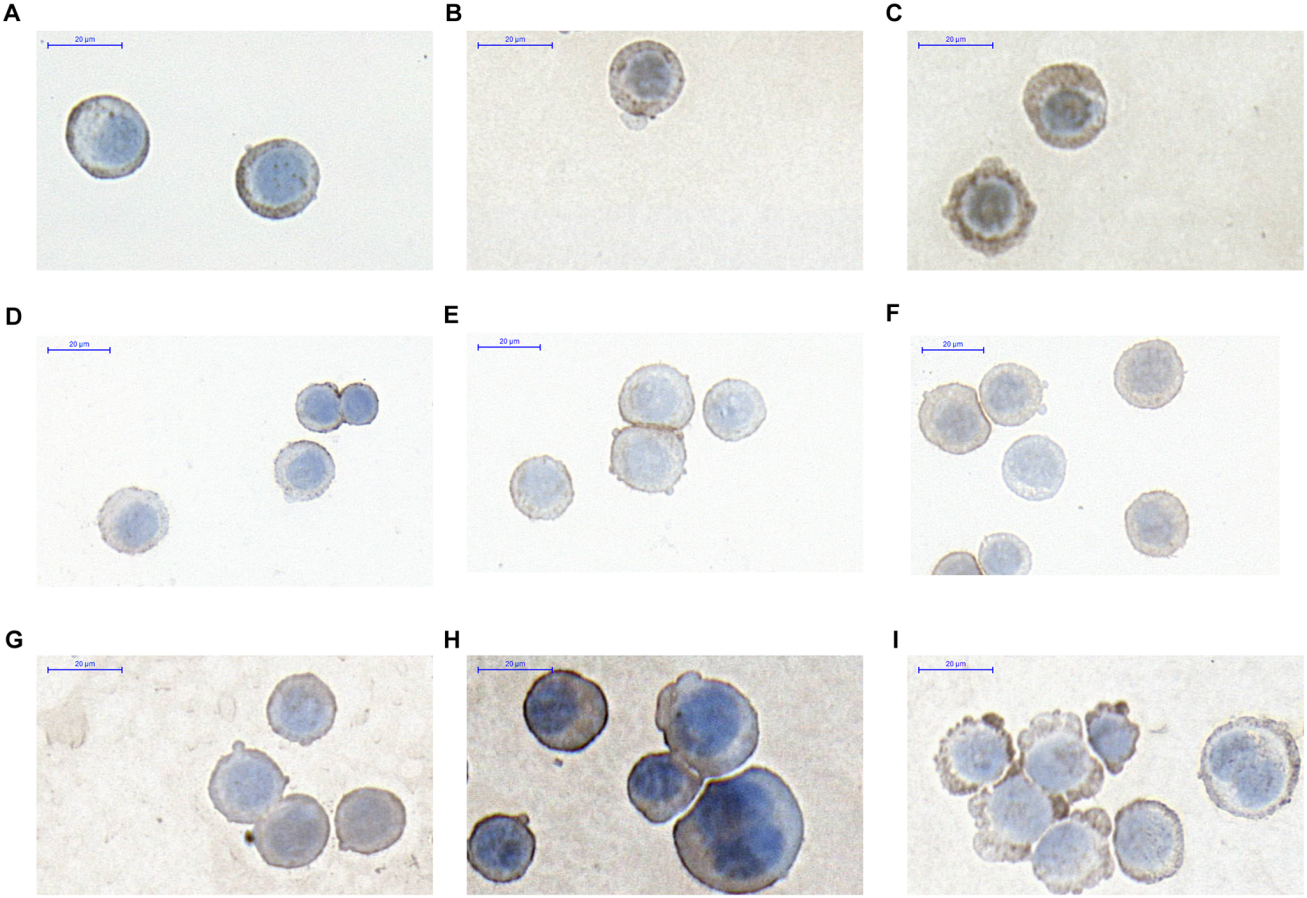
CK20 expression in HT-29 colon cancer cells after the treatment by sterile PBS(A), by DNA from normal colon epithelium (B), by DNA from tumorous colon epithelium (C) E-cadherin expression after the treatment by sterile PBS(D), by DNA from normal colon epithelium(E), by DNA from tumorous colon epithelium(F) DNMT3a expression after the treatment by sterile PBS(G), by DNA from normal colon epithelium(h), by DNA from tumorous colon epithelium(I).

Here we selected three epithelial markers i.e. CK20. E-cadherin, DNMT3a based on our previous results. [28] PBS treated HT-29 cells showed weak membrane CK20 (Fig 6A), E-cadherin (Fig 6D) and weak/moderate cytoplasmic DNMT3a (Fig 6G) protein expression. Tumor DNA treatment induced increase in expression of CK20 strong positive (+++) pixels, (Fig 6C) E-cadherin weak (+) and moderate (++) positive pixels (Fig 6F) and DNMT3a weak (+) positive pixels (Fig 6I). (p≤0.05). Tumor DNA promoted CK20 and E-cadherin expression in the non-differentiated, HT—29 colon adenocarcinoma cells both on mRNA and protein level (Table 4, Fig 6C and 6F) (p≤0.05). DNMT3a protein overexpression correlated with the previous results [28].

**Table 4:** Table representing RNA expression change of CDH1 and KRT 20 after normal and tumor tissue derived DNA treatment.

Probe Set ID	Gene Symbol	t-test (Control vs Normal DNA treatment)	t-test (Control vs Tumor DNA treatment)	Log FC (Normal DNA treatment vs Control)	Log FC (Tumor DNA treatment vs Control)
201131_s_at	CDH1	0,030924	0,004002	0,25845	0,735174
213953_at	KRT20	0,057182	0,007309	0,177712	0,741828

### ICC analysis of transcription factors and methylation markers in fibroblasts

**Fig 7:**
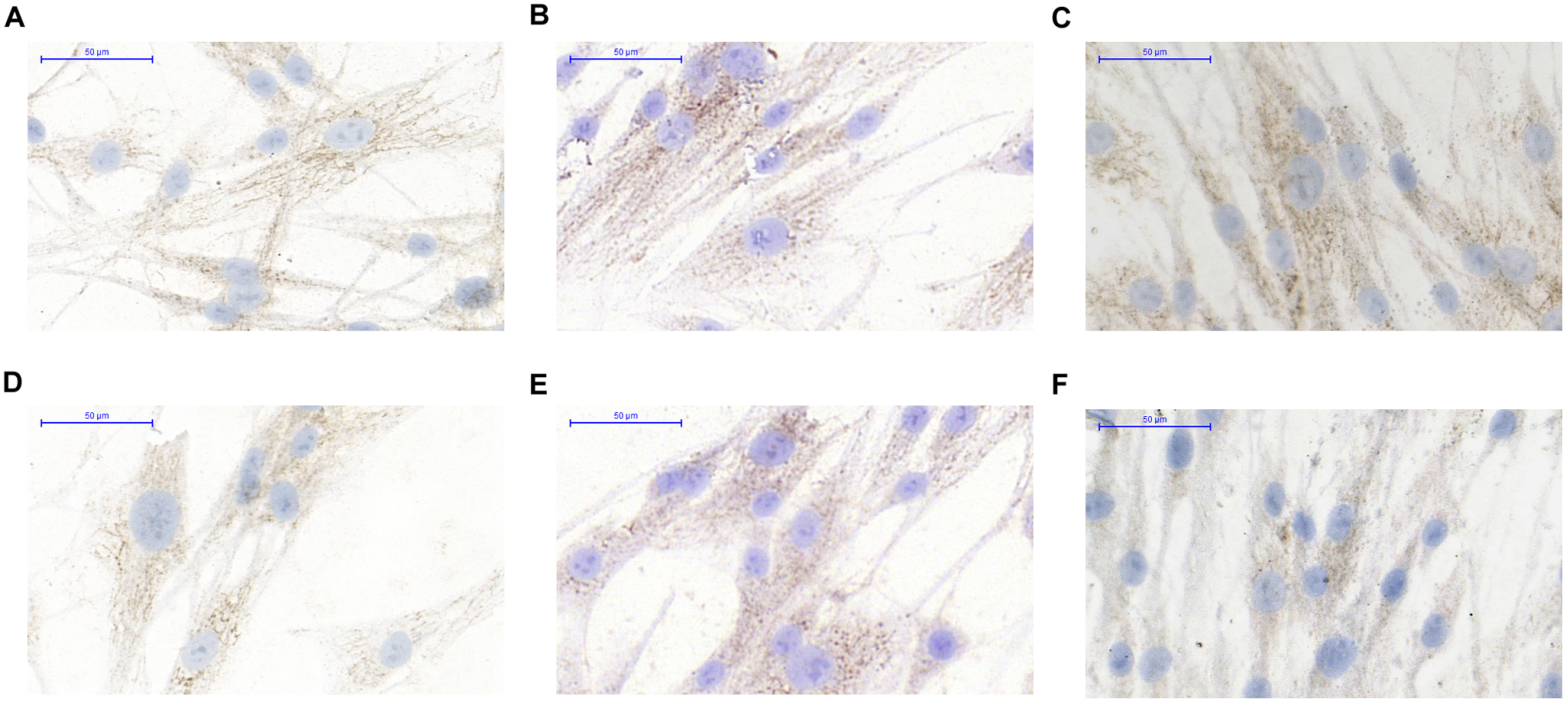
DNMT3a expression in HDF alpha fibroblasts after the treatment by sterile PBS(A), by DNA from normal colon epithelium(B), by DNA from tumorous colon epithelium(C), NFκB expression after the treatment by sterile PBS(D), by DNA from normal colon epithelium(E), by DNA from tumorous colon epithelium(F).

The NF-κB (p65 subunit) reflected weak cytoplasmic / nucleolic expression in PBS treated control fibroblasts (Fig 7D). By the effect of normal tissue DNA (Fig 7E), but not tumor tissue originated DNA, (Fig 7F) the fibroblasts reflected increase in expression of NFκB weak positive pixels (+).

The cytoplasmic DNMT3a expression was on a weak (+), moderate (++) level in normal fibroblasts (Fig 7A). The ratio of weak (+) positive pixels was slightly increased after normal and tumor DNA treatment (Fig 7B and 7C).

**Fig 8:**
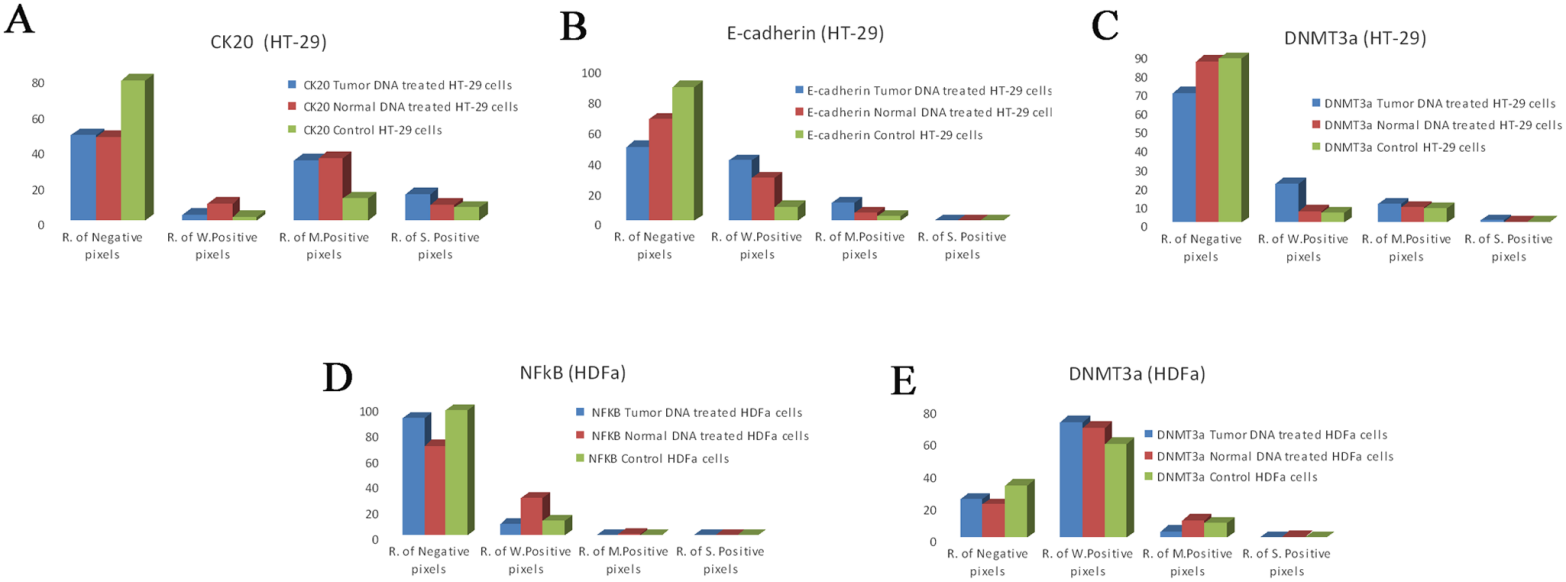
ICC evaluation of CK20(A), E-cadherin(B) and DNMT3a(C) on HT-29 colon cancer cells and NFκB(D) and DNMT3a(E) in HDF alpha fibroblasts.

Our immunocytochemical results on HT-29 cells and HDFα fibroblasts are summarized in the Fig 8A, 8B, 8C, 8D and 8E.

## Discussion

The main aim of our work was to examine changes in gene expression following administration of cell free DNA isolated from healthy and tumorous colonic tissue on HT-29 epithelial cells and fibroblasts. We are not aware of any studies that have examined expression changes in the two different cell types cooperating during tumorigenesis or the effect of cell free DNA from different origins. According to recent published studies, bacterial DNA activates DNA sensing receptors, possibly due to the frequency of unmethylated CpG sequences in bacterial genome, which is 20 fold higher than in vertebrates’ genome.[35] From further studies it was clear that DNA from pathogenic bacteria upregulate TLR9 expression [15], In the present study we have examined the gene expression changes induced by DNA of tumor origin in cancer cells, by whole genome array. This approach is similar to that reported by Lee et. al. (2014) who demonstrated the uptake, incorporation and effect of this tumor tissue originated DNA on cell proliferation.[13]

Our gene expression results are confirmed align with observations in recent publications that demonstrated that intact tumor DNA binds as a ligand to TLR9 but does not result in downstream activation of TLR9 pathway [36]. In our study, our whole genome array results provided evidence that tumorous DNA acts as a pro-metastatic factor independently from the TLR9 and STING pathways by inducing overexpression of metastasis-associated genes (MACC1, MALAT1,TACSTD2), the tumor marker (CEA), metabolic genes (INSIG1, LIPG) and messenger molecule genes (DAPP, CREB3L2). MACC1 is an important pro-metastatic factor that showed significant overexpression following treatment with tumor DNA. This gene is closely associated with the alterations of expressions of cell cycle- and invasion-related proteins.[37] Our results reflecting overexpression of other important pro—metastatic genes (MACC1, MALAT1, TACSTD2) are confirmed by previous data reflecting the major role of these genes in metastasis and their association with poor prognosis in various human epithelial cancers.[29, 37, 38]

Tumor tissue derived DNA treatment affected INSIG 1 and LIPG expression. These two key elements of endothelial lipid metabolism are also known to be associated with cancer progression. [31, 32]Increased LIPG expression was reported as a possible urinary cancer biomarker.[32] Studies described increased expression of secondary messenger molecules (DAPP, CREB3L2) in colon cancer with the growth and invasion of colon cancer cells via PI3K/AKT pathway activation.[33]

We observed that treatment with tumor DNA also results in overexpression of cytokeratin 20, and E-cadherin which was validated both at the RNA (Table 4) and the protein level (Fig 6C and 6F). E-cadherin as a very important factor regulating cell—cell interaction also showed higher expression in HT-29 cells treated with tumor DNA. E-cadherin overexpression leads to the suppression of β-catenin-Tcf/Lef-dependent transcription and likely inhibits cell apoptosis. [39]

DNA methylation changes mediated by DNMT3a are characteristic of colon cancers. Re-expression of factors targeting DNMT3a expression significantly decreased tumor growth. DNMT3a overexpression after treatment with tumor DNA infers a potential connection between the methylation status of tumor tissue derived DNA and the mechanism of action of tumor tissue derived DNA sequences acting through DNA sensing receptors. Former studies highlight a crucial role of this enzyme in colorectal cancer formation.[40]

Colon cancer tissue is not isolated, but with its cellular milieu through chemokines, cytokines, growth and apoptosis signals is communicating with other tissues. Studies have examined the role of the normal and cancer associated fibroblasts (CAFs) in colorectal cancer and showed that CAFs co-cultured with epithelial cells increases the tumor growth [41], but they do not determine the role of normal fibroblasts separately in tumorigenesis. Our aim was to determine the reaction of normal fibroblasts around the epithelial crypts to the released DNA and to screen the pathways included in that process.

In normal fibroblast treatment with DNA from normal tissue caused activation of the TLR9 canonic pathway, as qRT-PCR revealed overexpression of many genes involved in TLR9 MYD88 dependent pathway (MYD88, IRAK2, NFκB, IL-8, IL-1β).

From whole genome microarray analysis results we can conclude that healthy DNA in fibroblast is not only recognized by TLR9 MYD 88 dependent pathway, but we found that the elements of STING-dependent cytosolic pathway also showed significant overexpression, which initiates IFN production. Using the KEGG pathway map (http://www.genome.jp/kegg-bin/show_pathway?hsa04623+340061) 4 genes (ADAR, IRF7, CXCL10, CASP1) from the STING pathway showed significant overexpression after the treatment with healthy DNA in HDF-α fibroblasts. This activated cGAS/STING-dependent DNA sensing pathway induced overexpression of interferon regulatory factors IRF1, IRF2, IRF7, IRF9. and induced increased expression of interferon receptor genes (IFNAR2, IFNGR2). (log FC≥1, p≤0.05) The overexpression of CASP1 and NLRC 5 suggest NLRC5-dependent activation of the inflammasome. Davis et. al. published that NLRC5 cooperates with NLRP3and RNA interference-mediated knockdown of NLRC5 nearly eliminated caspase 1 activity. [42]

NFκB as a transcription regulatory factor showed significant overexpression in the fibroblasts treated by normal DNA. (Fig 7E) It shows that cell free DNA induced NFκB translocation regulating the release of pro-inflammatory cytokines and growth factors.

Whole genome microarray analysis showed a strikingly different number of genes regulating healthy and tumor tissue derived DNA administration (613 and 12 genes, respectively). While healthy DNA activated DNA sensing systems and upregulated many genes for cytokines, chemokines, growth factors, apoptosis related genes, prostaglandins, tumor DNA affected only few genes, that do not involve in this regulating processes. We think that normal fibroblasts do not react to tumor DNA and a long-term cancer-fibroblast interaction could initiate a transformation of normal fibroblasts to carcinoma-associated fibroblasts supporting “metastatic” phenotype.

Our study points the possible metastasis promoting effect of the tumor originated DNA via TLR9 independent pathway due to the overexpression of metastasis associated genes, metabolic genes and signaling pathways driving promoting cancer cell invasion. Differences in the physical properties of DNA isolated from tumor versus normal tissue suggests different mechanism of action in non-malignant and cancer cells through the activation of different pathways. Recently published works highlight that during the oncogenic transformation, in addition to changes in methylation status, other structural changes are observed, including changes in the expression of oncogenes and tumor suppressor genes, aberrant microsatellites, and rearranged chromosomal DNA incorporation to host cells genome.[13] Based on the tumor promoting effect the cf—DNA it´s degradation could be a possible therapeutical target achieved by DNAse treatment.[5]
